# Do sentences with unaccusative verbs involve syntactic movement? Evidence from neuroimaging

**DOI:** 10.1080/23273798.2014.887125

**Published:** 2014-03-21

**Authors:** Z.K. Agnew, H. van de Koot, C. McGettigan, S.K. Scott

**Affiliations:** ^a^ Institute for Cognitive Neuroscience, UCL, 17 Queen Square, LondonWC1N 3AR, UK; ^b^ Research Department of Linguistics, UCL, 2 Wakefield Street, LondonWC1N 1 PF, UK

**Keywords:** language, fMRI, syntax, temporal cortex

## Abstract

This study focuses on the neural processing of English sentences containing unergative, unaccusative and transitive verbs. We demonstrate common responses in bilateral superior temporal gyri in response to listening to sentences containing unaccusative and transitive verbs compared to unergative verbs; we did not detect any activation that was specific to unaccusatives. Our findings indicate that the neural processing of unaccusative and transitive verbs is highly similar, and very different from the processing of unergative verbs. We discuss the consequences of these results for the linguistic analysis of movement phenomena.

As is well known, natural language exhibits various kinds of ‘displacement’ phenomena, where constituents surface in a noncanonical position. Some examples are provided in Table 1, Appendix 1 the direct object of the transitive verb *hire* occupies its canonical position in (1a), but surfaces in a derived position in (1b–d). The silent category occupying the position in which the moved category originates is called the ‘trace’ of movement.

**Table:** 

(1)	a.	John hired this man	(canonical)
	b.	This man John hired this man	(focus movement)
	c.	Which man did John hire which man	(wh-movement)
	d.	I fired the man who John hired who	(relativisation)

**Table 1.** Example sentences of each condition.

Condition	Examples
Unergatives	The aides resigned a day into the scandal.
	The banks participated two years in a row.
Unaccusatives	Some friends arrived a week before the party.
	The rivers swelled the day after the rain.
Transitives	The thief abandoned the car after the crash.
	The inmates voiced objections to the new regime.

The sentences in (1b–d) all involve the so-called A′-movement, a syntactic operation which targets an A′-position in the left periphery of the clause. A′-positions are associated with discourse-related interpretive properties, such as topic and focus (see for example, Kiss, 1998; Reinhart, 1983; Rizzi, 1997), and are outside the inner clausal domain in which predicate–argument relations are established. By contrast, the so-called A-movement, illustrated in (2b and c), displaces a constituent to an agreeing or case-marked position (namely the subject position). Such positions, commonly referred to as A-positions, can potentially receive a *θ*-role and therefore do form part of the inner domain of the clause.

**Table:** 

(2)	a.	John sank the boat.	(canonical)
	b.	The boat was sunk the boat.	(passivisation)
	c.	The boat sank the boat.	(movement with unaccusatives^1^)
	d.	John seems John to have sunk the boat.	(raising)

The hypothesis that sentences with certain intransitive verbs involve movement (The Unaccusativity Hypothesis; Burzio, 1986; Perlmutter, 1978) is supported by a range of empirical evidence. We briefly review some of this evidence. First, in many languages unaccusatives distinguish themselves from unergatives in the auxiliary they select for the perfective (see Burzio, 1986 for Italian, Everaert, 1986 for Dutch). Second, unaccusatives, but not unergatives, can appear with resultative predicates (Levin & Rappaport-Hovav, 1995; Tsujimura, 1994; Van Voorst, 1986; see the contrast between 3a and 3b). Unaccusatives pattern in this respect with transitives (see 3c). Furthermore, the fact that resultative predicates with transitives are invariably object-oriented lends support to the idea that the subject of an unaccusative has been moved from object position. Third, participles of transitive verbs can be prenominal modifiers of nouns corresponding to their object (see 4a). This is also true of unaccusatives (see 4b), but not of unergatives (see 4c) (Hoekstra, 1984; Williams, 1981, among many others):

**Table:** 

(3)	a.*	Dora shouted hoarse.	(unergative)
	b.	The vase broke in pieces.	(unaccusative)
	c.	John painted the barn green/*tired.	(transitive)
(4)	a.	The damaged book.	
	b.	The recently arrived student.	
	c.*	The slept student.	

Fourth, unaccusatives have also been shown to display distinctive behaviour in acquisition (Pierce, 1989, 1992). Fifth, using a cross-modal lexical decision task, Friedmann, Taranto, Shapiro, and Swinney (2008) show reactivation of the subject NP in postverbal position in NP-V sentences containing unaccusatives but not in NP-V sentences containing unergatives. Finally, agrammatic aphasics are known to have difficulty with the production of sentences with unaccusative verbs (Kegl, 1995; Lee & Thompson, 2004, 2011), although comprehension is relatively spared (see Lee & Thompson, 2004; Piñango, 2000).

In virtually all work in the Chomskyan tradition, the displacements in (1) and (2) are reduced to a single syntactic operation, commonly referred to as Move. Recent work in the so-called Minimalist Programme (Chomsky, 1993, 1995, and much subsequent work) goes one step further and treats Move as a special instance of the more general binary operation Merge, which combines two trees into a single syntactic object. External Merge combines two unconnected trees into a connected tree (see Figure 1a), where *α* and *β* may be terminal nodes or trees, while Internal Merge (Move) combines a tree with a copy of a subtree taken from within it (see Figure 1b; we will henceforth refer to this as the ‘copy theory of movement’).

**Figure 1.**
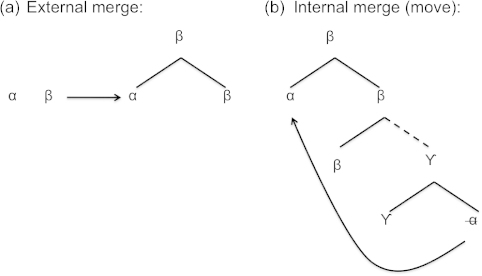
External and internal Merge.

If it is indeed the case that all movements involve the same syntactic operation, then one might reasonably expect to be able to detect a stable computational signature in psycholinguistic and neurolinguistic experiments investigating structures with A- and A′-movement, respectively. As a matter of fact, however, the experimental record is far from straightforward in this regard.

It has been reported in studies using cross-modal lexical priming (CMP) that traces of A′-movement trigger priming of their antecedent at the gap location (Love & Swinney, 1996; Nakano, Felser, & Clahsen, 2002). CMP experiments have also found clear differences between sentences with unergative and unaccusative verbs, with only the latter exhibiting priming for the surface subject in a position following the verb, an effect that may be plausibly attributed to the presence of a postverbal trace created by A-movement (Friedmann et al., 2008; Osterhout & Swinney, 1993). But the timing of the priming effect found differs substantially; with passives and unaccusatives it is found some 750 ms downstream from the gap location. This is a robust finding that has recently been replicated for unaccusatives in an eye-tracking study using the visual world paradigm (Koring, Mak, & Reuland, 2012).

Neurolinguistic studies probing brain activation associated with movement have focused primarily on A′-movement (Ben-Shachar, Hendler, Kahn, Ben-Bashat, & Grodzinsky, 2003; Ben-Shachar, Palti, & Grodzinsky, 2004; Santi & Grodzinsky, 2007, 2010). These studies converge on the finding that A′-movement requires involvement of the left inferior frontal gyrus. In addition, A′-movement has also been found to activate superior temporal regions (Ben-Shachar et al., 2004; Just, Carpenter, & Keller, 1996; Röder, Stock, Neville, Bien, & Rösler, 2002). In recent work, Shetreet, Friedmann, and Hadar (2010) investigate the neural processing of Hebrew sentences with unaccusative verbs, comparing it to the neural processing of sentences with unergative and transitive verbs, and report significantly greater activity in left IFG during perception of sentences with unaccusative verbs compared to sentences with unergative or transitive verbs. Shetreet & Friedmann, 2012 have replicated the findings for unaccusative verbs in a study comparing them to unergative and reflexive verbs.^2^ The activation in anterior left inferior frontal gyrus (LIFG) found by Shetreet and colleagues for sentences with unaccusative verbs tallies with earlier functional magnetic resonance imaging (fMRI) studies focusing on passives in Japanese and Mandarin (Kinno, Kawamura, Shioda, & Sakai, 2008; Ye & Zhou, 2009; Yokoyama et al., 2006, 2007), which all found frontal activation^3^.

Since the specific area activated in sentences with unaccusative verbs, left IFG, is also selectively activated by A′-movement, one could begin to speculate that the stable computational signature for syntactic movement that has proved so elusive in psycholinguistic work has now been successfully identified at the neural level.

The present study is a variant of the studies by Shetreet et al. (2010) and Shetreet and Friedmann (2012), but with the important difference that none of the verbs used in the experiment has a plausible source from which it can be derived. All unergative and unaccusative verbs used in the experiment lack a transitive counterpart, while all the transitive verbs lack an intransitive counterpart. This restriction to non-alternating verbs has two key advantages.

First of all, the cross-modal priming study reported in the work of Friedmann et al. (2008) found consistent postverbal reactivation of the subject of non-alternating unaccusatives. By contrast, alternating unaccusatives displayed mixed properties, with some displaying the reactivation pattern of non-alternating unaccusatives and others behaving like unergatives in not showing any reactivation at all. This finding suggests that using non-alternating unaccusatives provides the best opportunity for detecting neural activations due to A-movement.

Second, while it is of course possible that non-alternating unaccusatives are derived from transitive sources that are themselves not words of the language, it could very well be that they lack such a source altogether. If so, language processes would use a non-alternating unaccusative accessed in the lexicon in much the same way they would use an unergative, namely ‘as is’. As a result, if our study were to confirm activation in LIFG specific to sentences with unaccusatives, then this activation may be attributed to A-movement with somewhat greater confidence. However, if no such activation were to be found, it would lend some support to the view that the activation in LIFG identified in previous studies of passives and unaccusatives could be associated with a process responsible for the suppression of the external argument, or with additional semantic processing triggered by the absence of the argument.

The study reported below uses functional MRI to isolate neural activation specific to the A-movement involved in sentences with non-alternating unaccusative verbs. We do this by comparing the processing of such sentences with very similar sentences containing non-alternating unergative and transitive verbs not involving this operation. In these ways, our experimental design parallels that of Shetreet et al. (2010; see also Shetreet & Friedmann, 2012); however, our use of a passive design, with no overt task required, allows us to reduce the possibility of task-related neural activity. Thus any neural differences in the processing of the different sentence types must be attributed to differences in the underlying syntactic representations, rather than to aspects of the requirement to process this information overtly (Hickock, 2009). Furthermore, the English sentences used in the experiment lack any surface cues that would allow a hearer to distinguish sentences containing an unergative verb from sentences containing an unaccusative verb.^4^ This makes it very unlikely that any top-down influences would have affected the outcome of this experiment.

## Materials and methods

### Generation of stimuli

Subjects were exposed to sentences containing an unergative, unaccusative or transitive verb. All sentences used in the experiment had the general form NP V Det N PP, where the Det-N-PP sequence was a modifier in the sentences containing unergative and unaccusative verbs, while in sentences containing a transitive verb N was the head noun of the direct object, with the following PP being a constituent of either the NP or the VP.

The three sets of stimuli were matched for syllable count and for frequency of the predicate (using the British national Corpus). Sentences were recorded in an anechoic chamber and saved as.*wav* files. The sound files were normalised using the peak amplitude in Praat (Boersma & Weenink, 2010) and low pass filtered at 4 kHz. Sentences were performed by a native British speaker who produced 30 tokens for each category of verb. Rotated versions (Blesser, 1972) were generated by spectrally inverting a selection of the original sentences. This transformation provides a high-level acoustic baseline, which is not intelligible, but retains the temporal and spectral complexity of the original speech. Thus the final five conditions were sentences containing unergative verbs, sentences containing unaccusative verbs, sentences containing transitive verbs, rotated versions of these sentences, and a silent baseline condition. The three groups of sentences were matched for syllable number (means = Unergatives: 11.57, Unaccusatives: 11.79, Transitives: 11.93). Mean pitch and spectral centre of gravity were measured using Praat (Boersma & Weenink, 2010). One-way ANOVAs were conducted to compare mean and standard deviation of pitch and spectral centre of gravity (spectral centre of gravity measures the distribution of signal intensity across the spectrum). Mean pitch was found to be significantly different across conditions [*F*(2, 81) = 8.755, *p* < 0.005]; post hoc tests revealed that this was due to the mean pitch being significantly greater for the transitive than the unergative sentences. There was no significant effect of any of the other measures across the three sentence types (Pitch SD [*F*(81, 2)=4.974, *p* = 0.009], spectral centre of gravity [*F*(81,2)=3.060, *p* = 0.052], SD of spectral centre of gravity [*F*(81,2)=2.811, *p* = 0.66]).

#### Subjects

Seventeen healthy right-handed subjects (11 female, mean age 25.3 yrs ± 3.5) participated in the present study. All gave informed consent according to the guidelines approved by UCL Ethics Committee, who provided local ethics approval for this study.

#### Scanning

A 1.5-Tesla Siemens system was used to acquire 183 T_2_*-weighted echo-planer images (EPI) data (3 × 3 × 3 mm^3^, TR/TE/flip 10,000 ms/50 ms/90°) using BOLD contrast. A SPARSE scanning protocol was employed in order to administer the auditory stimuli in silence. The first two functional volumes were discarded in order to remove the effect of T_1_ equilibration. High-resolution T_1_ anatomical volume images were also acquired for each subject.

#### fMRI

During the main experimental run, subjects lay supine in the scanner in the dark and were asked to close their eyes and listen to the sentences played to them. They were not told that the sentences differed in any way. There was no other task involved, so as to avoid any form of neural activity that a response task might entail (such as motor priming in response to a button-press task or higher-order processing associated with working memory or error monitoring). Each event was formed of one sentence administered through headphones (Etymotic, IL, USA) lasting a maximum of 3 seconds. There were 28 examples of each condition played in a randomised order with ±500 ms onset jitter. This main run lasted approximately 30 minutes.

#### Pre-processing and analyses

Functional data were analysed using SPM8 (Wellcome Department of Imaging Neuroscience, London, UK) running on Matlab 7.4 (Mathworks Inc, Sherborn, MA, USA). All functional images were realigned to the first volume by six-parameter rigid body spatial transformation. Functional and structural (T_1_-weighted) images were then normalised into standard space using the Montreal Neurological Institute (MNI) template. Functional images were then coregistered to the T_1_ structural image and smoothed using a Gaussian kernel of full width half medium (FWHM) 8 mm. The data were high-pass filtered at 128 Hz. First-level analysis was carried out using motion parameters and all four acoustical measures (mean and standard deviation of pitch, mean and standard deviation of spectral centre of gravity) as regressors of no interest at the single-subject level. A random-effects model was employed in which the data were thresholded at *p* < 0.005. Voxelwise thresholding was carried out at 30 voxels to limit potential type II errors.

Individual contrasts were carried out to investigate the BOLD response to each condition minus the silent rest or rotated speech. Contrasts were thresholded at *p* < 0.005 with a cluster threshold of 30 voxels, uncorrected. A null conjunction (Nichols, Brett, Andersson, Wager, & Poline, 2005) was employed in order to look at regions significantly activated in more than one condition. This approach takes the intersection mask of two thresholded images so that it is possible to look at voxels that are significantly active in the contrast (A > B) and also in the contrast (C > D). These were carried out using a masking threshold of *p* < 0.001. Significant effects in the conjunction tell us that there are common activations in two conditions; however, failing to find significant effects in the conjunction is not evidence of no effect.

Significant BOLD effects were rendered on a normalised template. For the purpose of additional anatomical precision, group contrasts were overlaid on a surface-based representation of the MNI canonical brain using the SPM surfrend toolbox (written by I. Kahn; http://spmsurfrend.sourceforge.net). Local foci of maximal activation were then identified using cytoarchitechtonic and probabilistic atlases (Eickhoff et al., 2005). Region of interest analysis was carried out to investigate mean effect sizes in specific regions (5-mm-diameter spheres) across all experimental conditions against baseline, using the MarsBar toolbox available for use within SPM8 (Brett, Anton, Valabregue, & Poline, 2002).

## Results

**Figure 2.**
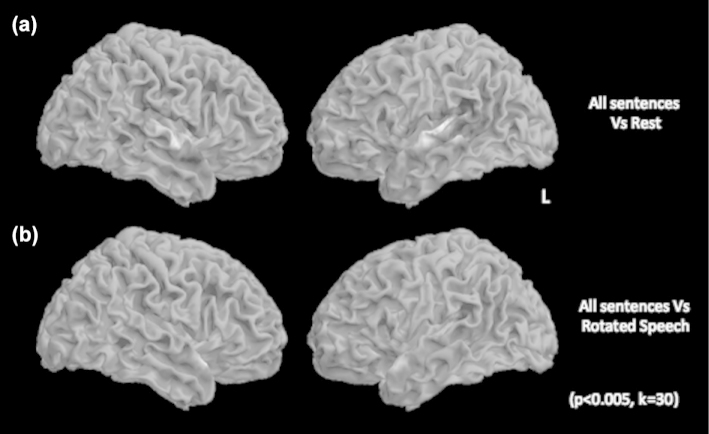
Passive perception of speech is associated with widespread activity in bilateral dorsolateral temporal lobes. A comparison of perception of all sentences compared to a rest condition was associated with significant activity in bilateral dorsolateral temporal cortices, extending from posterior to middle/ anterior STG and inferior frontal gyrus in the left hemisphere (a). A comparison of all speech perception conditions with a high-level baseline of (unintelligible) rotated speech revealed significant activity in bilateral mid/anterior STG in both hemispheres, premotor cortex and extra-striate visual areas in the left hemisphere (b). Activity in STG in this contrast spread anteriorly compared to the speech vs. rest condition.

**Figure 3.**
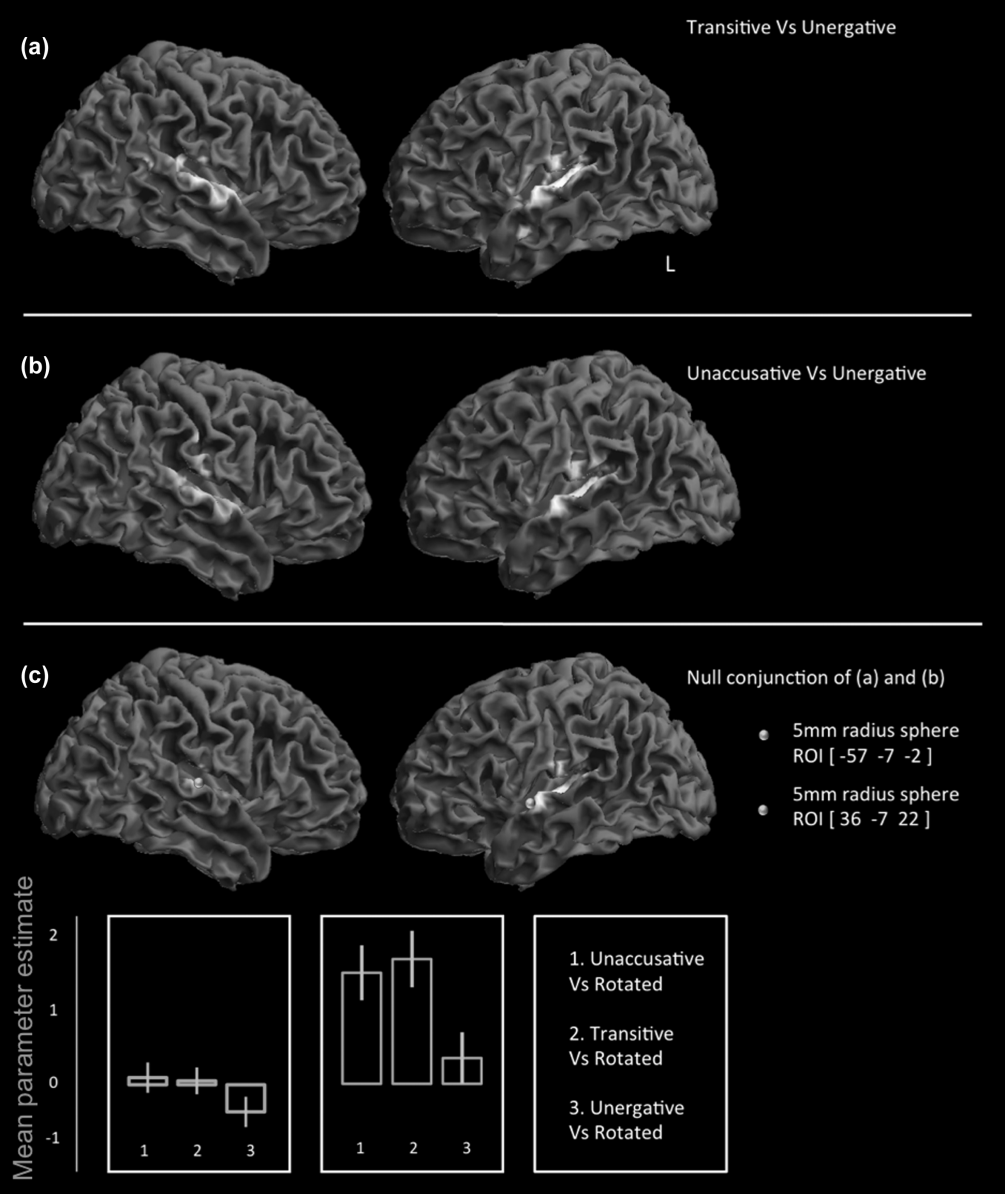
Perception of sentences with unaccusative and transitive verbs is associated with activity in similar areas, compared to perception of sentences with unergative verbs. For the comparison of sentences with transitive and unaccusative verbs, mean parameter estimates were extracted from spherical regions of interest of 5-mm radius, taken from peak coordinates from the null conjunction of (unaccusative vs. unergative) and (transitive vs. unergatives). The null conjunction revealed peak coordinates in superior temporal gyri in both hemispheres (*p* < 0.005 uncorrected) shown in (c). The lines denote the cortical activations during perception of unaccusative vs. unergative and transitive vs. unergative. Both peaks lay in superior mid-temporal gyri. Parameter estimates for three contrasts are plotted for both of these region of interest (ROI)s. In both regions there was greater activity during perception of unaccusative and transitive sentences.

Passive perception of all sentences over silent rest was associated with widespread activity in bilateral dorsolateral temporal lobes (Figure 2a) consistent with other studies (Scott, Blank, Rosen, & Wise, 2000; Scott, Rosen, Lang, & Wise, 2006; Wise, Greene, Buchel, & Scott, 1999). Comparison of sentence perception with the high-level baseline of rotated speech revealed activity in more anterior regions of the superior temporal gyrus and encompassed additional activation in inferior frontal gyrus, inferior posterior parietal cortex and striate visual cortex in the left hemisphere (Figure 2b). Perception of each verb type separately contrasted against rotated speech produced comparable maps of activation, comprising dorsolateral temporal cortices in both hemispheres, although to a much greater extent in the left, encompassing anterior and posterior superior temporal gyrus and extending into inferior frontal gyrus and dorsolateral prefrontal cortex. In the right this activity was restricted to anterior superior temporal gyrus. A direct comparison of the verb types revealed that unaccusative and transitive verb perception compared to unergative verbs was associated with significant activity in middle and posterior superior temporal gyri in both hemispheres (Figure 3a and b). A null conjunction of these two contrasts confirmed common activity in middle and posterior superior temporal gyri in both hemispheres (Figure 3c).

In order to probe this activity further, a region of interest analysis was carried out by extracting the mean activity from 5-mm-diameter spheres at the peaks generated from the null conjunction of (unaccusative vs. unergative) and (transitive vs. unergative) contrasts. These peaks are shown in Figure 3c. For both peak locations, activity is less for perception of unergative sentences, and equivalent for transitive and unaccusative sentences. In the left superior temporal gyrus the response to unergative sentences is below baseline (i.e. there is a greater response to rotated speech than to unergative verb containing sentences in the left peak).

In order to compare the current data to those from Shetreet et al. (2010) and Shetreet and Friedmann (2012), we looked at the activity specific to the processing of unaccusative verbs. To this end, we carried out a null conjunction of the contrasts (unaccusative > unergative) and (unaccusative > transitive). This did not reveal any significant activity.

## Discussion

The present study aimed to isolate neural activity specific to the processing of sentences with unaccusative verbs, as opposed to sentences with unergative or transitive verbs, by looking at passive perception of these sentence types. We report significant activity in bilateral dorsolateral temporal cortices, encompassing middle and posterior superior temporal gyri in both hemispheres, and ventral parietal operculum/primary motor cortices on the right, during perception of sentences with unaccusative and transitive verbs, but not for sentences with unergative verbs.

These results demonstrate not only that the neural processing of both unaccusative and transitive verbs is significantly different to that of unergative verbs, but that the former two both elicit similar spatial patterns of activity in the superior temporal gyrus. As such, they present a double analytical challenge.

First, what would explain the apparent similarity of sentences with unaccusative and transitive verbs, and what sets these verbs types apart from unergatives?

Second, our experiment has not clearly replicated the findings of Shetreet et al. (2010). In particular, we have failed to find any activation that is specific to the neural processing of sentences with unaccusative verbs. Given the otherwise rather copious empirical support for an A-movement analysis of unaccusatives, this presents us with something of a conundrum; we expect to find some computational signature of A-movement, but in fact we find no such thing. How can this finding be explained?

Before we explore other possibilities, let us begin by asking whether our results could not simply reflect the aspectual properties of our test items. As is well known, properties of both verbs and their internal arguments play a role in determining the telicity of a predicate (Verkuyl, 1972). In our experiment, this fact could potentially have contributed significantly to the common processing of transitives and unaccusatives, since they are sentences with these verb types that are most likely to show telic aspect, whereas sentences with unergatives are by and large atelic. There are however a number of reasons why we can be confident that telicity is not the main driver behind our results.

First, there was a very significant difference in the proportion of telic items in the set of transitive sentences (50%) and the set of unaccusative sentences (100%). If the shared activation between these sentence groups was largely due to aspectual processing, then we should have found a significant difference, contrary to fact.

Second, if aspectual processing is responsible for the differences we found between unergatives, on the one hand, and transitives and unaccusatives, on the other hand, then it follows that telic aspect is associated with increased activity in superior temporal gyrus (STG) according to our data. However, Husband and Stockall (2012) demonstrate using two self-paced reading experiments that it is the processing of sentences with atelic aspect that seems to incur the higher processing cost, which is not in alignment with the pattern of activity that we report here.

Third, most experimental work on aspect has focused on aspectual coercion, the phenomenon where a VP-modifier (verb position) forces a repetition reading for a telic predicate (as in *John jumped over the fence for two hours*; see Piñango, Winnick, Ullah, & Zurif, 2006 and references cited there). In a MEG study of this phenomenon, Brennan and Pylkkänen (2008) find a two-stage effect, which they claim is most easily interpreted as reflecting a right-lateral detection of anomaly followed by a prefrontal meaning shift. The right-lateral anomaly detection is located by them in the right anterior temporal lobe. Suppose we assume that the anomaly detection is in the region where aspect is actually processed (although Brennan and Pylkkänen themselves make no such claim), then this would allow the inference that the right anterior temporal lobe is involved in aspectual processing. While the shared activation associated with transitive and unaccusative sentences found in our study does indeed extend to this region, its primary focus lies in middle and posterior superior temporal gyri and occurs bilaterally.

Finally, we are aware of one recent experimental result that locates the detection of telic aspect in the left posterior middle temporal gyrus (Romagno, Rota, Ricciardi, & Pietrini, 2012). These investigators specifically look at the effect of telicity within various regions of interest identified by perception of verb containing sentences, and they find no significant difference between telicity and atelicity in any regions of STG or superior temporal sulcus (STS).

**Figure 4.**
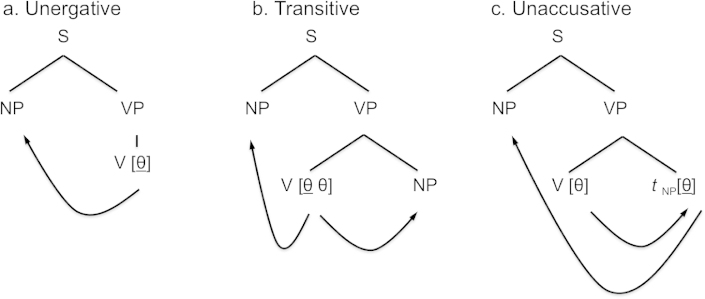
The syntactic structures projected from unergative, transitive and unaccusative verbs; blue arrows indicate *θ*-marking relations.

We conclude that, on balance, it is unlikely that our results are a reflection of the aspectual properties of our test items. But if it is not aspect, then what else could explain the apparent similarity of sentences with unaccusative and transitive verbs? And what sets these verb types apart from unergatives? There is, in fact, precisely one analysis of sentences with unaccusative verbs that makes them look rather similar to transitives and that is the theory of ‘theta-role transmission’ first put forward by Williams (1987) (see also Williams, 1994; see Neeleman & Van de Koot, 2002, 2010 for related discussion). Williams suggests that the trace of A-movement is a predicate: it transmits the thematic role it receives to the NP that is its antecedent, as shown in Figure 4c, where *t*_NP_ stands for the unpronounced copy of the moved subject NP and blue arrows represent relations of *θ*-marking.

What the theta-role transmission theory amounts to is the claim that a single semantic role (let's say the theme) is assigned to the subject of an unaccusative in two steps; it is first assigned to the trace in object position and subsequently transmitted to the subject of the unaccusative verb. On this analysis, sentences with a transitive verb (Figure 4b) and those with an unaccusative verb both exhibit two instances of theta-role assignment. In transitives, one *θ*-role is assigned to the object of the sentence and the other to the subject. In sentences with an unaccusative verb, a *θ*-role is assigned to the race of the subject in object position, and this same *θ*-role is subsequently assigned to the subject itself, via *θ*-role transmission. By contrast, sentences with an unergative verb (Figure 4a) contain a single *θ*-role assignment, namely to the subject.

On the simplest assumption, the assignment of a *θ*-role, no matter whether that *θ*-role originates in a verb or in a trace, involves activity in bilateral posterior and middle superior temporal gyri. Therefore, the *θ*-role transmission theory correctly predicts that unaccusatives should pattern with transitives, rather than with unergatives, as regards the magnitude of activation resulting from establishing thematic relations.

By contrast, our findings are problematic for the view that the relation between the subject of an unaccusative verb and the postverbal trace is the same as that established in A′-movement relations. On that analysis, unaccusative verbs require an application of this unitary movement operation that is absent in sentences with unergative or transitive verbs. Therefore, the processing of such sentences should show activation not found with sentences containing unergatives and transitives. In fact, it should plausibly be accompanied by activation in brain regions previously claimed to subserve A′-movement, such as LIFG and superior temporal regions (Ben-Shachar et al., 2003, 2004; Just et al. 1996; Röder et al., 2002; Santi & Grodzinsky, 2007, 2010). Of course, our experiment did find activation in superior temporal regions, but this was present with unaccusatives and transitives in equal measure so that it cannot be said to identify a property unique to unaccusatives.

Furthermore, again on the view that the relation between the trace and the subject in sentences with unaccusative verbs is identical to the relation established in A′-movement relations, the level of activation associated with *θ*-marking in unergatives and unaccusatives should be similar, and this level should be lower than that found with transitives. But, as we have seen, this prediction is not borne out.

Our finding that the magnitude of activation resulting from establishing thematic relations is considerably lower for unergatives is consistent with previous work that has demonstrated that verb processing in general is associated with activity in bilateral posterior temporal parietal regions (Devauchelle, Opheim, Rizzi, Dehaene, & Pallier, 2009; Friederici, Ruschemeyer, Hahne, & Fiebach, 2003) and that this activity is sensitive to increasing argument structure complexity. For example, Shapiro, Gordon, Hack, and Killackey (1993) found evidence that the retrieval of a verb's thematic properties is mediated by dedicated resources that show load effects directly correlated with thematic complexity. Ben-Shachar et al. (2003) and Thompson et al. (2007) report effects of transitivity in the lateral posterior superior temporal sulcus (LpSTS)/STG/middle temporal gyrus (MTG). Finally, den Ouden, Fix, Parrish, and Thompson (2009) reported that production of transitive verbs compared to intransitive verbs was associated with activity in posterior temporal cortex and left inferior cortex. Whilst this is a production study, rather than a perception study it is likely that perception and production of syntax may rely on common processing to some extent.

We now turn to the second challenge presented by our results: why did our study not find any activation specific to the neural processing of sentences with unaccusative verbs, in contrast to the studies reported in Shetreet et al. (2010) and Shetreet and Friedmann (2012)? We believe that the answer may lie in the fact that we restricted our study to non-alternating unaccusatives.

It is commonly assumed that unergative verbs are retrieved from the lexicon without lexical derivation. By contrast, the majority of unaccusative verbs are thought to undergo derivation from their transitive counterpart through suppression of their external cause/agent argument (Chierchia, 1989; Reinhart, 2002; Reinhart & Siloni, 2005). Reinhart (2002) refers to the relevant operation as expletivisation reduction. While passives differ from unaccusatives in important respects, they, too, undergo an operation that prevents their external argument from being projected into the syntax.^5^ In short, then, A-movement in passives and unaccusatives is made possible by the fact that these verbs do not realise an external argument. These two properties, suppression of an external argument and A-movement, go hand-in-hand.

Now recall that none of the unaccusative verbs used in the present study has a plausible transitive source from which it could have been derived, and it seems plausible that they lack such a source altogether. If so, language processes would use a non-alternating unaccusative accessed in the lexicon in much the same way they would use an unergative, namely ‘as is’, without the need for suppression of an external argument. This being so, it is conceivable that the frontal activation found with studies using alternating unaccusatives and passives is an effect on the suppression of the external argument rather than of the A-movement that it enables.

In summary, the results obtained in the experiment reported here can be made compatible with the well-motivated A-movement analysis of unaccusatives provided such movement is analysed as involving *θ*-role transmission. This would allow one to infer that the computational signature of A-movement might differ substantially from that of A′-movement and may in fact be more akin to the *θ*-marking relation. This non-unitary character of what linguists assume to be a single, unitary ‘movement’ relation might then also be held responsible for previous failures to detect a uniform effect of syntactic movement in cross-modal priming experiments.

## Conclusion

The present work investigated the structural representation of sentences containing unaccusative verbs, in which the subject is associated with a trace in object position through a process known as A-movement. Our primary aim was to investigate the relation between the subject and the trace in such sentences and to establish whether this relation gives rise to signature neural processing.

The results of our study strongly suggest that the neural processing of sentences with unaccusative verbs is rather similar to that of sentences with transitive verbs and very different from the processing of sentences with unergative verbs. This finding supports the conclusion that A- and A′-movements should not be analysed as a unitary syntactic operation. More specifically, the pattern of neural activation for sentences with unaccusatives reported here provides strong support for the view that the subject in such sentences is related to its trace through a process of *θ*-role transmission and that *θ*-role assignment, no matter whether a *θ*-role originates in a verb or a trace involves activity in bilateral dorsolateral temporal cortices.

## Notes

1. Intransitive verbs come in two types. The so-called unergatives have a subject that is interpreted as the initiator of the action it expresses (*The man* *smiled*), while the so-called unaccusatives have a subject that is interpreted as a patient (or theme) – the argument that undergoes the action expressed by the verb, such as *the boat* in example (2c). Like the initiator of a transitive verb (such as *hit*), the initiator of an unergative is structurally realised as a subject. The single patient argument of an unaccusative is realised as the object of the unaccusative verb, but surfaces as its subject due to movement (as illustrated here).

2. The design of this study also successfully addressed a confounding factor in that of Shetreet et al. (2010) which left open the possibility that the activity in IFG associated with unaccusatives was due to the morphological marking that is found on Hebrew unaccusatives.

3. See also Christensen (2008) for evidence for activation in the left anterior temporal cortex with Danish data involving operator movement in the IP domain.

4. NP-V sentences containing unaccusatives can be distinguished prosodically from NP-V sentences containing unergatives (see Adger, 2007). However, this variation is eliminated by the addition of a postverbal modifier (as is the case in our stimuli containing these verb types).

5. There are, however, important differences between passives and unaccusatives. On Reinhart's view, reduction in unaccusatives is a radical operation: it literally removes the argument from the verb's lexical semantics (Reinhart, 2002). By contrast, the suppressed argument in passives can still licence purpose clauses and the like.

## Appendix

**Unergatives**: hesitate, operate, work, smile, wave, bounce, resign, sleep, scream, participate, laugh, differ, argue, listen.

**Unaccusatives**: exist, arrive, evolve, vanish, die, swell, slip, rise, depart, occur, emerge, remain, fall, disappear.

**Transitives**: abandon, review, exceed, receive, voice, supply, attach, modify, admired, suggest, create, replace, avoid, accept.
